# Efficacy of Alendronate for Preventing Collapse of Femoral Head in Adult Patients with Nontraumatic Osteonecrosis

**DOI:** 10.1155/2014/716538

**Published:** 2014-11-11

**Authors:** Yu-Cai Hong, Ru-Bin Luo, Tiao Lin, Hui-Ming Zhong, Jian-Bin Shi

**Affiliations:** ^1^Department of Emergency, Sir Run Run Shaw Hospital, School of Medicine, Zhejiang University, No. 3 East Qingchun Road, Hangzhou 310008, China; ^2^Department of Emergency, Research Institute of Emergency Medicine, Second Affiliated Hospital, School of Medicine, Zhejiang University, No. 88 Jiefang Road, Hangzhou 310009, China; ^3^Department of Orthopaedic Surgery, Second Affiliated Hospital, School of Medicine, Zhejiang University, No. 88 Jiefang Road, Hangzhou 310009, China

## Abstract

The purpose of the current review was to determine the efficacy of alendronate for preventing collapse of femoral head in adult patients with nontraumatic avascular osteonecrosis of femoral head (ANFH). Five randomized controlled trials (RCTs) involving 305 hips were included in this review, of which 3 studies investigated alendronate versus control/placebo and the other 2 studies compared the combination of alendronate and extracorporeal shockwave therapy (ESWT) with ESWT alone. Our results suggested that even the patients with extensive necrosis encountered much less collapse in the alendronate group than control group. In these RCTs, their data also indicated a positive short- and middle-term efficacy of alendronate treatment in joint function improvement and hip pain diminishment. With the presence of the outlier study, only insignificant overall efficacy of alendronate could be observed with substantial heterogeneities. In addition, we did not find any additive benefits of alendronate in combination with ESWT for preventing collapse compared to ESWT alone. In conclusion, there is still lack of strong evidence for supporting application of alendronate in adult patients with nontraumatic ANFH, which justified that large scale, randomized, and double-blind studies should be developed to demonstrate the confirmed efficacies, detailed indication, and optimized strategy of alendronate treatment.

## 1. Introduction

Osteonecrosis or avascular necrosis of femoral head (ANFH) is a disabling clinical disease that affects 20,000 persons each year in the United States. The progressive disease is characterized by reduced local blood flow and death of the osteocytes and the bone marrow [1]. During bone repair process, the predominant resorption of necrotic bone exceeding bone formation frequently leads to a progressive destruction of bone architecture, subchondral fracture, extensive hip pain, and loss of joint function. Ultimately, after collapse of femoral head, a standard total hip arthroplasty (THA) is indicated [2, 3]. Because of the young age of many of these patients, a hip replacement cannot be expected to last the patient's lifetime and a second surgery would be required. In addition, prior invasive treatments or periprosthetic infection, aseptic prosthesis loosening after THA commonly are attributed to the increased possibilities and difficulties in the following revision surgery. Therefore, when feasible, attempts should be made to save the femoral head prior to collapse with use of less invasive treatment modalities [4–6].

To identify such noninvasive treatment options with potential benefits becomes extremely desirable. Pharmacologic agents, which have been used to treat osteonecrosis of the hip are statins [7, 8], anticoagulants [9, 10], prostacyclin [11, 12], and bisphosphonates (Bps) [13–22]. The theoretical benefit of statins is based on the association of increased fat cell size with an increased risk of the development of osteonecrosis of the hip [23, 24]. Anticoagulants inhibit the aggregation of platelets and enhance blood flow to ischemic areas of bone [10]. Prostacyclin promotes bone regeneration on a cellular or systemic level but fail to show efficacy in the advanced stages of ANFH [11]. Up to now, there has been no consensus with regard to the ideal treatment for the precollapse stage of ANFH. In contrast to other drugs, Bps are potent antireabsorptive agents that act by inhibiting the action of mature osteoclasts in the bone, which theoretically normalized the uncoupled bone remodeling contributing to femoral head collapse [20]. In the last decade, many studies therefore investigated the application of Bps in the treatment of ANFH [13–22]. Biophysical means, including extracorporeal shock-wave therapy (ESWT) [25–27] and electromagnetic therapy [28–30], have shown to be effective for early ANFH due to the increased ingrowth of neovascularization and new bone formation. Since these noninvasive procedures have different working mechanism from Bps, the synergic efficacies of two treatments, like ESWT combined with oral Bps, were also tested in recent studies [31, 32].

Nevertheless, the lack of controlled groups, the substantial heterogeneities in genres in Bps, population of patients (adults and juvenile), and etiology of necrosis (traumatic and nontraumatic cases) complicated the interpretation of the recent systematic reviews [20, 21] and necessitated new evidence.

In the current review, only randomized controlled trials consisting of adult patients with nontraumatic osteonecrosis using alendronate alone or in combination with any other physical therapies were included, from which the data extracted were synthesized in meta-analysis manner. The rationales were as follows: (1) application of BPs in juvenile raised great debates due to its potential harmful effects of the growing skeleton; (2) alendronate is second-generation nitrogen-containing Bps with potent antireabsorptive effects [33], which is also the most widely prescribed Bps for this population [13–22]; (3) traumatic ANFH has a distinct progress pattern from nontraumatic cases and most of traumatic patients commonly need surgical intervention [34]; (4) pooled data from RCTs rather than descriptive summary of uncontrolled trials could provide evidences with higher quality for clinical practice or future research.

Therefore, by summarizing the latest relevant randomized controlled trials, the purpose of the current meta-analysis and systematic review was to determine the efficacy of alendronate alone or in combination with other biophysical modalities for adult patients with nontraumatic ANFH. Our hypothesis was that alendronate therapy in this population would be well tolerated and (1) retard the collapse progression of femoral head, (2) improve clinic function and hip pain, (3) be more effective if combined with other biophysical treatments.

## 2. Methods

### 2.1. Literature Search

Electronic databases (Pubmed, EMBASE, and the Cochrane Central Register of Controlled Trials) were searched without limit by two independent investigators (H.Y.C. and L.T.), which were updated in August 2014. The search used terms and Boolean operators as follows: “(conservative treatments or alendronate or Fosamax) and (avascular necrosis or aseptic necrosis or osteonecrosis) and (femoral head).” There was no limitation on language, year of publication, or publication status. We manually searched reference lists of review articles and included studies to identify other potentially eligible studies as well.

### 2.2. Identification of Eligible Studies

Trials were included if they contained all of the following: (1) the study was randomized controlled trial; (2) the study exclusively targeted adult patients with nontraumatic ANFH; (3) the treatment was alendronate alone or alendronate in combination with any other physical therapies; (4) the study provided at least with adequate data on retardation of bone collapse of femoral head. After exclusion of duplicates, 2 reviewers (L.R.B. and L.T.) performed an initial title and abstract screening of articles to discard those that were clearly ineligible; then, 2 reviewers (H.Y.C., Z.H.M.) independently examined the full article to assess the trials for eligibility for inclusion, with disagreements resolved by discussion. Citations were excluded if (1) they were noncontrolled clinical trials or animal studies; or (2) they consisted of adolescences and/or were treated with other Bps; or (3) they combined alendronate with invasive procedures to treat ANFH. If necessary, we attempted to contact the author of the original report to obtain further details.

### 2.3. Assessment of Study Quality

Two reviewers (H.Y.C. and L.R.B.) independently assessed the study validity with Cochrane Collaboration's tool for assessing the risk of bias, which addresses five specific domains such as randomization schedule, allocation concealment, blinding, selective outcome reporting, and follow-up rate [35]. Whether the included trials were similar in baseline and adopting similar cointerventions were also evaluated. In addition, the level of evidence of each study was rated on basis of* Oxford Centre for Evidence-based Medicine—Levels of Evidence (March 2009)* [36]. Disagreement was resolved by discussion [35].

### 2.4. Data Abstraction, Conversion, and Analysis

From each article we extracted the following details: authors, year of publication, and geographical location of study, study design, study population (hips/patients), patient gender/age, stage of ANFH, detection of ANFH, dosage and duration of alendronate treatment, timing of alendronate initiation, and follow-up duration by using standardized forms.

Femoral head collapse is the common indication for hip arthroplasty, which is therefore not individually summarized and discussed in this review. The outcome of our interest primarily focused on the collapse of the femoral head after alendronate treatment. For the radiographic evaluation, although various classification systems were applied among studies, they shared fundamental similarities and therefore collapse rates were considered as a new occurrence of collapse or an increased collapse of greater than 2 mm [4, 37]. Those data across the inclusion studies were pooled and summarized estimates of treatment effect as risk ratio (RR) with 95% confidence intervals (CI) using the Mantel-Haenszel method. We also assessed the inconsistency *I* ^2^ to describe the percentage of the variability in effect estimates due to the heterogeneity. We considered a value of *I* ^2^ greater than 50% as the substantial heterogeneity. Fixed effects model would be applied if there were no statistical heterogeneity among the studies; otherwise, we used the random effects model [35].

We could not carry out funnel plots analysis due to insufficient trials included in our review. We performed post hoc sensitivity analysis by omitting the outlier studies from the main meta-analysis to determine their contribution to Cochran's heterogeneity in the overall analysis [38]. The outliers were defined as the studies with confidence interval of the estimated effect size not overlapping with the pooled overall effect size. After the identification of outlier, we presented and discussed our results separately with or without the outlier(s).

Stratified failure rate was analyzed, in which the extracted follow-up data were investigated after two new stratification groups being made for analysis, including Group 1: precollapse with extensive necrotic area (>30%, termed as C type of ARCO stage I–III or Steinberg stage I–III); Group 2: other precollapse. In the current review, only group 1 was applicable for meta-analysis as there was not sufficient data available in group 2.

The Review Manager (RevMan 5.3) software program (The Nortic Cochrane Centre, Copenhagen, Denmark, provided by The Cochrane Collaboration) was used for graphical representation of the pooled data.

The other outcomes of interest included clinical function, hip pain improvement, and adverse events associated to alendronate treatments. These data could not be analyzed using a meta-analysis due to the heterogeneities and limited number of the available RCTs.

## 3. Results

### 3.1. Study Identification

**Figure 1:**
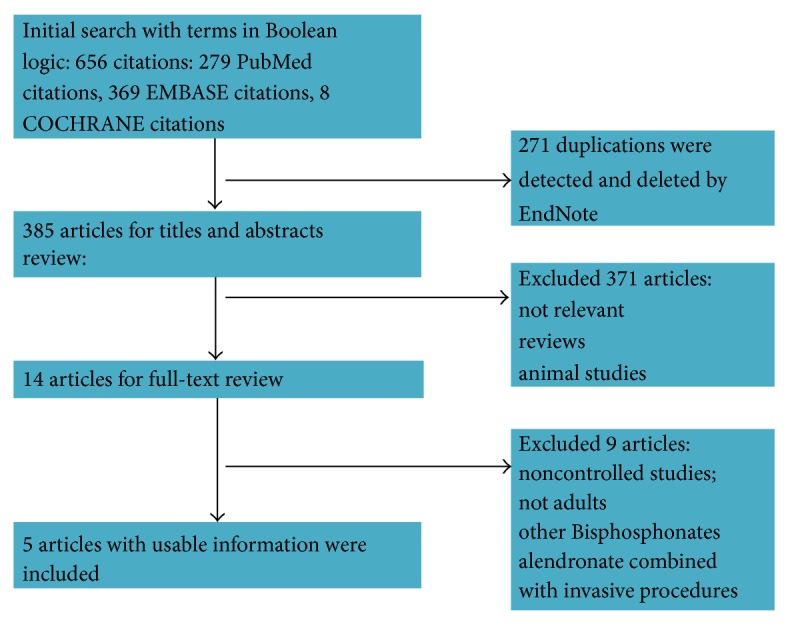
A flow diagram demonstrates the method of article selection for clinical study inclusion.

Figure 1 detailed articles identification, inclusion, and exclusion. Our search strategy initially yielded 85 citations. Of these, we included 5 RCTs with 305 hips in this systematic review [13–19, 22].  Table 1 shows the characteristics of the included articles. All studies were undertaken in Asia published in the recent 10 years with follow-up of 12–48 months, of which, 3 studies investigated alendronate alone versus control/placebo [13–15] and the other 2 studies compared the combination of alendronate and ESWT versus ESWT alone [31, 32]. No RCTs were found to apply the combination of alendronate with other physical therapies. The dosage, timing of initiation, and duration of alendronate administration varied among the studies. The included studies exclusively targeted adult nontraumatic ANFH patients, most of whom were caused by chronic usage of steroid or alcohol. The studied patients were all within stage III based on X-ray and/or Magnetic resonance imaging (MRI) according to Steinberg (University of Pennsylvania staging system, 2 studies) [13, 14] or Association Research Circulation Osseous (ARCO staging system, 3 studies) [15, 31, 32].

### 3.2. Validity Assessment

The methodological quality was evaluated independently by two reviewers (Z.H.M. and L.R.B.) with Cochrane Collaboration's tool for assessing the risk of bias summarized in Table 2 [35]. Two trials [13, 32] described adequate randomization, proper blinding, which were low risk of bias, while the other three trials with inexplicit randomization and inadequate blinding were considered moderate risk of bias [14, 15, 31]. Overall, the level of evidence for the mentioned studies ranged from 1b to 2b [36].

### 3.3. Rate of Collapse

**Figure 2:**
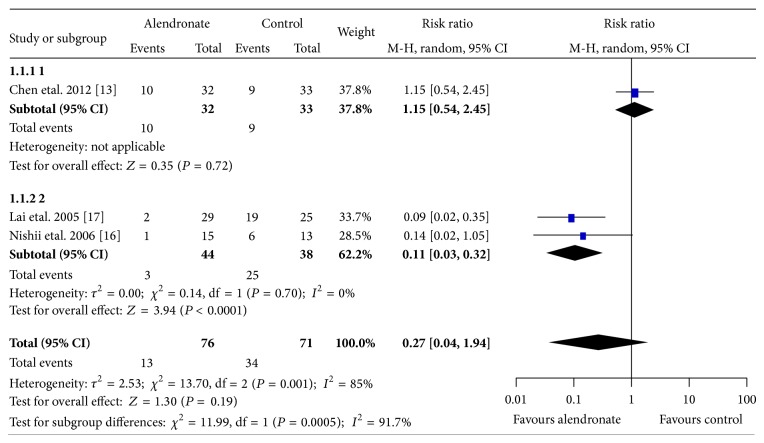
Graph showing comparing collapse rates prevention between alendronate and control groups in all the included patients. Chen's study was found to be the outlier as its confidence interval of the estimated effect size did not well overlap with the pooled overall effect size. Without the outlier, the overall effect favours alendronate over control (*P* < 0.0001) with minimal heterogeneities (*I* ^2^ = 0%). The size of each square is proportional to the weight of the study. Z: *P* value of weighted test for overall effect, CI: confidence interval, df: degree of freedom, *I* ^2^ test statistic.

As an end-point of follow-up, all of the five included studies reported the collapse rate. Meta-analysis was performed using the five studies (305 hips were followed) [13–15, 31, 32].  Figure 2 showed the results comparing alendronate alone versus control. The overall results showed substantial inconsistences and Chen's study was found to be the outlier which contributed 100% to the heterogeneities [13]. The pooled data omitting Chen's study demonstrated a significant reduction of collapse in the alendronate group than the control group (2 studies [14, 15]; alendronate: 3/44, 7%; control: 25/38, 66%; RR 0.11; 95% CI 0.03–0.32; *P* < 0.0001) and the heterogeneities were minimal (*I* ^2^ = 0%), while Chen's study presented comparable collapse rate between the alendronate and the control group (1 study [13]; alendronate: 10/32, 31%; control: 9/33, 27%; RR 1.15; 95% CI 0.54–2.45; *P* = 0.72), leading to the insignificant overall effect sizes of alendronate with substantial heterogeneities (3 studies, alendronate versus control, RR 0.27; 95% CI 0.04–1.94; *P* = 0.19; *I* ^2^ = 85%).

**Figure 3:**
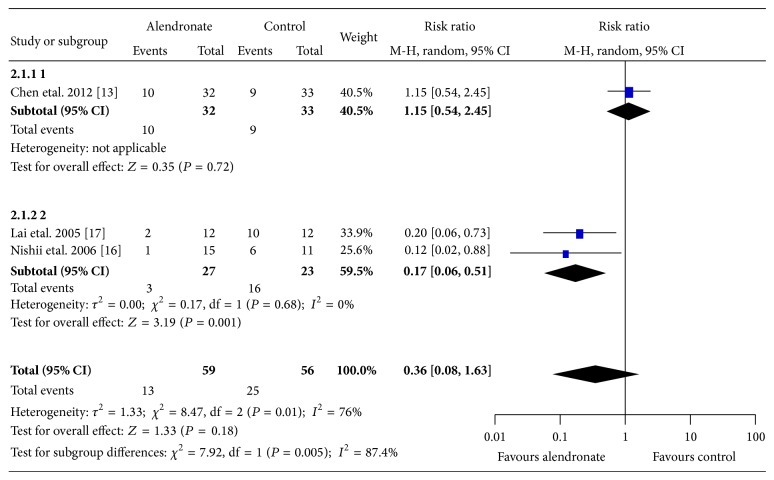
Graph showing comparing collapse rates prevention between alendronate and control groups in patients with extensive necrotic lesion (>30%). Chen's study was found to be the outlier as its confidence interval of the estimated effect size did not well overlap with the pooled overall effect size. Without the outlier, the overall effects favour alendronate over control (*P* = 0.01) with minimal heterogeneities (*I* ^2^ = 0%). The size of each square is proportional to the weight of the study. Z: *P* value of weighted test for overall effect, CI: confidence interval, df: degree of freedom, *I* ^2^ test statistic.

In cases of femoral head with extensive necrotic area (>30%), the efficacy of alendronate for preventing collapse was also found to be only significant with the absence of Chen's study (2 studies [14, 15]; alendronate: 3/27, 11%; control: 16/23, 70%; RR 0.17; 95% CI 0.06–0.51; *P* = 0.001; *I* ^2^ = 0%; showed in Figure 3).

**Figure 4:**
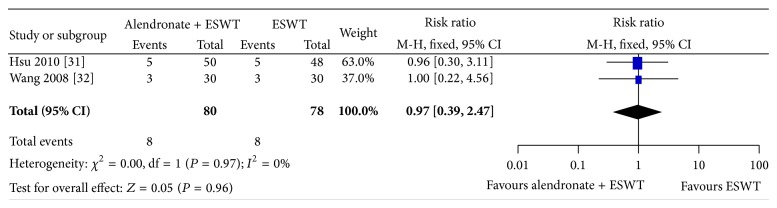
Graph showing comparing collapse rates prevention between alendronate plus extracorporeal shockwave therapy (ESWT) and ESWT alone groups. The overall effect was similar in the both groups (*P* = 0.97) with minimal heterogeneities (*I* ^2^ = 0%). The size of each square is proportional to the weight of the study. Z: *P* value of weighted test for overall effect, CI: confidence interval, df: degree of freedom, *I* ^2^ test statistic.

In the comparison of alendronate + ESWT versus ESWT alone, we did not found any additive benefits of alendronate in combination with ESWT for preventing collapse compared to ESWT alone (2 studies [31, 32], combined treatments: 8/80, 10%; ESWT alone: 8/78, 10%; RR 0.97; 95% CI 0.39–2.47; *P* = 0.96; *I* ^2^ = 0%; showed in Figure 4).

### 3.4. Clinical Outcome Score

Four studies were found to use Harris Hip Score (HSS) to evaluate the outcome [13, 14, 31, 32] (Table 3). In the outlier RCT performed by Chen et al. [13], no differences could be seen between alendronate and control group (alendronate: 79.3 ± 14.2; control: 83.8; *P* > 0.05) after 24-month follow-up. However, at the end of Lai's study, the HHS was 74.4 ± 7.8 points in the alendronate group, which was greatly higher than that in the control group (HHS: 49.2 ± 9.2 points). Only comparable results between ESWT+ alendronate versus ESWT were showed in other two studies [31, 32]. Specially, 18–48 months after the initiation of treatment, Hsu's study demonstrated the HHS was 87.8 ± 8.4 points in the ESWT+ alendronate group while that is 90.8 ± 12.9 in the EWST group [31]. Similarly, 95.3 ± 8.0 points of HSS in ESWT+ alendronate group and 94.3 ± 4.5 points in ESWT group were seen in the study of Wang et al. at the end of 12-month follow-up [32].

### 3.5. Hip Pain

Only three studies reported data of hip pain improvement [15, 31, 32] (Table 3). The study comparing alendronate with control conducted by Nishii et al. [15] demonstrated the grade of hip pain was unchanged in 6 hips and worsened in 7 hips in the control group. In contrast, the grade of hip pain in the alendronate group was unchanged in 15 hips, worsened in 1 hip, and improved in 4 hips. The other two studies [31, 32] consistently indicated patients from ESWT groups achieved significant pain reduction after treatment (ESWT alone: Hsu's study: hip pain reduced from VAS 5.4 ± 2.0 to 1.1 ± 1.5; Wang' study: from VAS 5.97 ± 2.30 to 0.6 ± 1.06) but the addition of alendronate only achieve the same magnitude of hip pain relief (ESWT + alendronate: Hsu's study: hip pain reduced from VAS 5.4 ± 2.2 to 1.5 ± 1.3; Wang' study: from VAS 5.03 ± 2.75 to 0.69 ± 1.19).

### 3.6. Adverse Events Analysis

None of the studies noted serious adverse effects related to alendronate administration. The most common side effects across the studies were gastric dyspepsia that were mentioned in 2 studies [15, 31] (Table 3), which occurred after treatment initiation and were self-limiting. No osteonecrosis of the jaw or atypical fractures were seen irrespective of the dose/duration of alendronate.

## 4. Discussion

Many surgical procedures have been described for preventing femoral collapse and progression of ANFH, such as nuclear decompression, osteotomies, nonvascularized bone grafts, and vascularized grafts [4, 39]. Nevertheless, due to the reported efficacy of total hip arthroplasty and the typical age group in those patients with osteonecrosis, it has recently been questioned whether these invasive procedures are appropriate, given the potential difficulty of later conversion to a hip replacement [4, 40].

Conservative treatment which helps improve function improvement and delay femoral head deformity could be valued buy-time strategy in those population. As we noted in literature search, there were two recent systematic reviews evaluating BPs for ANFH, one of which only included 3 observational short-term studies for juvenile [20], and the other one with 6 small short-term trials encountered substantial heterogeneities across studies in patients group (adults and adolescence, nontraumatic and traumatic ANFH) and treatments (mixture of alendronate and other Bps treatment) [21]. The above heterogeneities and the majority of uncontrolled studies massively challenged the interpretation of their results.

Generally speaking, the studies included in the current review still present various limitations, such as small sample size of insufficient RCTs with short-term follow-up; different ANFH stage of patients when treatment initiated; lack of uniformity in dosage, initiation time, and duration of alendronate used.

Bearing in mind the above-mentioned limitations, up to now, the current review is the first systematic review exclusively included RCTs on this topic and summarized results in meta-analysis manner. We further restricted our inclusion criteria to RCTs that studied alendronate treatment for adult patients with nontraumatic ANFH. We only analyzed alendronate because it was the most widely studied BPs for ANFH. Due to ongoing debate on alendronate's long-term effects on the growing skeleton chosen, we limited the studied population to adults. The exclusion of traumatic ANFH further reduced heterogeneities across included studies. Moreover, we also separately analyzed the efficacy of alendronate in the patients with extensive necrotic area, which was reported to substantially affect the prognostic outcomes of ANFH [41]. The most distinct aspect of the current review is, after determining Chen's study was the major source of heterogeneities, the collected data were further stratified by presence or absence of the very study [13].

Collapse of the femoral head appears to be a consequence of the noncoupling of bone reabsorption and bone regeneration rates. Alendronate sodium is characterized pharmacologically by the ability to inhibit bone resorption by binding to bone mineral and subsequently inhibiting the activity of the osteoclasts [20]. Part of the osteoclast inhibiting action of alendronate is mediated through an action on osteoblasts [42]. In this context, collapse could be prevented if bone resorption was suppressed or slowed by alendronate until the formation of sufficient new bone [20].

Agarwala and his colleagues, in their report of 395 hips at a mean follow-up of 4 years (1–8 years), reported a radiographic progression to collapse in 12.6% (27 of 215 hips) in stage I and 55.8% (72 of 129 hips) in stage II (Ficat and Arlet staging) following treatment with alendronate 10 mg daily for 3 years [17, 18]. The same author in a recent publication of 53 hips at 10-year follow-up reported a 29% collapse rate in the precollapse stage of ON (10 of 34 hips) following 3 years of continuous alendronate use at 70 mg weekly [16]. The investigators thus concluded that the natural history of untreated ON with more than 70% collapse rate was favorably altered with alendronate use [16].

In accordance with previous data, our results implied even the patients with extensive necrosis encountered much less collapse in the alendronate group than control group. In those RCTs, their data also indicated a favorable short-term and middle-term efficacy of alendronate treatment in improvement of articular function and hip pain diminishment [14, 15]. In addition, there were no sever adverse effects associated with alendronate treatment observed in all the included studies. Nevertheless, one point should be noted that some of the patients treated with alendronate still failed to preserve the femoral head and were subjected to THA, which indicated the treatment could slow or delay but not totally prevent the occurrence of femoral head failure.

In Chen's study, no advantages were observed by alendronate treatment compared to placebo group and the investigators thought that the study was underpowered to detect statistical significance despite a numerical reduction in the rate of disease progression in the alendronate group [13]. Actually, the distinct aspect of Chen's study from other studies was the uncommon low incidence of femoral head progression in the placebo-treated group even though all the patients had a large necrotic lesion (>30%) [13]. Earlier studies reported an overall clinical progression rate of 77% to 98% in untreated ANFH hips at an average of 3-year follow-up [41, 43–45]. To sharply contrast with this, only 27% (9/33) occurred in Chen's placebo group with 24-month follow-up [13]. However, the authors did not give sufficient discussion and explanation regarding this. Therefore, the negative results from Chen' study should be treated with great caution even if it was a multicenter, randomized, double-blinded trial.

Extracorporeal shock-wave therapy (ESWT) [25–27] and electromagnetic therapy [28–30] were reported to be effective for treatment of early ANFH. The rationale for the use of ESWT or electromagnetic therapy rested on that they potentiated the healing process by stimulating neovascularization and new bone formation. Therefore, it is believed that additional treatment with such physical therapies may further improve the bone quality of the femoral head and improve the clinical result. In the current review, the additional treatment with alendronate of ESWT was effective in improving hip pain and other clinical results but was only comparable to those treated with ESWT alone. The concurrent application of alendronate with ESWT and short-term follow-up were considered to be the potential reason in both articles [31, 32]. On the other hand, it is important to recognize local bioavailability of alendronate for an avascular bone condition is impaired [20, 46], which lead to an insignificant synergetic effects of alendronate combined with other conservative treatment.

## 5. Conclusion

In conclusion, there is still lack of strong evidence for supporting application of alendronate in adult patients with nontraumatic ANFH. Large scale, randomized, and double-blind studies should be developed to demonstrate the following aspects. (1) The efficacy of alendronate for ANFH during long-term follow-up should be confirmed. (2) The detailed indication of ANFH for alendronate treatment should be further clarified; for example, what type of ANFH, traumatic or nontraumatic, which stage of ANFH, including what size and what location of the necrotic lesion, should be preferentially indicated. (3) Moreover, there are a number of patient-specific factors that must be considered, including age, comorbidities and life expectancy, health, and activity level. (4) We also need to optimize the strategy of treatment, including timing of treatment initiation and the dose and duration of alendronate therapy.

## Figures and Tables

**Table 1:** Characteristics of the included randomized controlled studies.

Author/year/area	Study design	Number of hips/patients		Gender (F/M)		Average age (year)		Stage of ANFH (*n*)		Detection of ANFH	Dosage/average duration of treatment	Combined with other treatments	Timing of treatment initiation	Average follow-up (month)
		Aln	Control	Aln	Control	Aln	Control	Aln	Control					
Chen et al. (2012) Taiwan [13]	Placebo-controlled RCT	32/26	33/26	4/22	7/19	48.4 ± 11.4	44.2 ± 9.2	Upenn stage II C (20), Stage III C (12),	Upenn stage II C (25), Stage III C (9),	MRI	70 mg per week for 104 weeks	NA	Aln: 0.9 ± 0.9 months; Placebo: 2.0 ± 2.9 months after diagnosis	24

Lai et al. (2005) Taiwan [14]	RCT	29/20	25/20	5/15	5/15	42.6 (22–65)	42.4 (20–64)	Upenn stage II (13); III (12)	Upenn stage II (12); III (3)	X-ray and MRI	70 mg per week for 25 weeks	NA	NA	24–28

Nishii et al. (2006) Japan [15]	RCT	20/14	8/13	7/7	7/1	48 (29–75)	36 (18–54)	ARCO: stage I (10); II (5); III (5)	ARCO: stage I (4); II (3); III (7)	MRI	5 mg per day for 1 year	NA	Within 4 years after diagnosis	12

Hsu et al. (2010) Taiwan [31]	RCT	50/28	48/35	10/18	8/27	39.1 ± 12.6	39.6 ± 11.9	ARCO: stage I (2); II (35); III (13)	ARCO: stage I (2); II (27); III (20)	X-ray and MRI	70 mg per week for 1 year	ESWT +/− HBO	Aln + ESWT + HBO: 6–48 months; ESWT: 6–18 months after onset of symptoms	18–48

Wang et al. (2008) Taiwan [32]	RCT	30/25	30/23	5/20	10/13	38.6 ± 12.6	35.7 ± 4.7	ARCO: stage I, II (19); III (11)	ARCO: stage I, II (25); III (6)	MRI	70 mg per week for 1 year	ESWT	Aln + ESWT: 6–18 months; ESWT: 6–20 months after onset of symptoms	12

Aln: alendronate; ANFH: avascular necrosis of femoral head; RCT: randomized controlled trial; ESWT: extracorporeal shock wave treatment; HBO: hyperbaric oxygen therapy; MRI: magnetic resonance imaging; ARCO: Association Research Circulation Osseous; Upenn: University of Pennsylvania System (Steinberg); NA: not available.

**Table 2:** Methodological quality of included randomized controlled trials.

Study	Randomized adequately^a^	Allocation concealed	Blinding^b^	Balance in baseline	Advoiding selective reporting	Similar cofactors	Follow-up rate	Level of evidence^d^
Chen et al. 2012 [13]	Yes	Yes	Double blinded	Yes	Yes	Yes	81%	1b
Lai et al. 2005 [14]	Unclear	No	No	Yes	Yes	Yes	100%	2b
Nishii et al. 2006 [15]	Unclear	No	No	Yes	Yes	Yes	88%	2b
Hsu et al. 2010 [31]	Yes	Yes	No	Yes	Yes	No^c^	93%	2b
Wang et al. 2008 [32]	Yes	Yes	Double blinded	Yes	Yes	Yes	92%	1b

^a^The trials which randomization schedules were explicitly described could get an “Yes”.

^b^The trials which placebo was adequately decribed how to blind both patients and investigators were considered as “Double Blinded”.

^c^In Hsu's study, they compared alendronate + extracorporeal shock wave treatment + hyperbaric oxygen therapy versus extracorporeal shock wave treatment alone.

^d^The level of evidence was rated on basis of Oxford Centre for Evidence-based Medicine-Levels of Evidence (March 2009) [36].

**Table 3:** Other outcomes reported from studies evaluating efficacy of alendronate in avascular necrosis of femoral head.

Study	Clinical function (HHS)				Hip pain (VAS)				Adverse effects
	Baseline		After treatment		Baseline		After treatment		
	Aln	Control	Aln	Control	Aln	Control	Aln	Control	
Alendronate versus control/placebo									
Chen et al. 2012 [13]	78.1 ± 12.5;	76.6 ± 15.2	79.3 ± 14.2	83.8 ± 12.8	NA	NA	NA	NA	None

Lai et al. 2005 [14]	67.6 (26–88);	65.7 (34–84)	74.4 ± 7.8;	49.2 ± 9.2	NA	NA	NA	NA	NA

Nishii et al. 2006 [15]	NA	NA	NA	NA	NA	NA	NA	Aln versus control unchanged: 15 versus 6, improved: 4 versus 0, worsened: 1 versus 7 (*P* = 0.003)	Allergy and abdominal discomfort: 2

Alendronate + ESWT versus ESWT alone									
Hsu et al. 2010 [31]	74.5 ± 10.8;	77.2 ± 14.5	87.8 ± 8.4	90.8 ± 12.9	5.4 ± 2.2	5.4 ± 2.0	1.5 ± 1.3;	1.1 ± 1.5	Dyspeptic symptoms: 3

Wang et al. 2008 [32]	79.2 ± 12.9;	75.1 ± 6.1	95.3 ± 8.0;	94.3 ± 4.5	5.03 ± 2.75;	5.97 ± 2.30	0.69 ± 1.19;	0.6 ± 1.06	None

Aln: alendronate; ESWT: extracorporeal shockwave treatment; HHS: Harrris hip score; VAS: visual analog scale; NA: not available.
